# Auriculotherapy for the intervention effect of chronic heart failure: a systematic review and meta-analysis

**DOI:** 10.3389/fcvm.2025.1685507

**Published:** 2026-01-08

**Authors:** Zhifang Luo, Lin Wang, Lei Yao, Xiaoyan Wen

**Affiliations:** 1 School of Basic Medical Sciences/School of Nursing, Chengdu University, Chengdu, China; 2 Zhujiaqiao Community Health Service Center, Pudong New Area, Shanghai, China; 3 Department of Urology, Affiliated Hospital of Chengdu University, Chengdu, China

**Keywords:** auriculotherapy, chronic heart failure, complementary therapy, meta-analysis, randomized controlled trials, systematic review

## Abstract

**Background:**

Auriculotherapy is a complementary therapy known to improve quality of life in various diseases; however, its clinical application in the treatment of chronic heart failure (CHF) remains limited, partly due to the lack of standardized efficacy evaluation indicators.

**Methods:**

Relevant randomized controlled trials on CHF were systematically searched from database inception to November 2025. Two reviewers independently screened studies, extracted data, and assessed study quality. Any disagreements were resolved by consensus with the assistance of a third reviewer. Meta-analyses were conducted using RevMan 5.4.

**Results:**

A total of 24 studies involving 2,387 patients were included in the study. The results of meta-analysis demonstrated that auriculotherapy combined with conventional treatment improved the effective rate of cardiac function improvement [odds ratio (OR) = 3.92, 95% confidence interval (CI): 2.86–5.38, *P* < 0.00001], increased left ventricular ejection fraction [mean difference (MD) = 4.07, 95% CI: 3.57–4.58, *P* < 0.00001], reduced left ventricular end-diastolic diameter (MD = −4.27, 95% CI: −4.98 to −3.56, *P* < 0.00001), and prolonged 6-min walking test distance (MD = 65.11, 95% CI: 62.55–67.68, *P* < 0.00001). It also reduced the Minnesota Living with Heart Failure Questionnaire score (MD = −7.29, 95% CI: −8.52 to −6.06, *P* < 0.00001). The incidence of adverse reactions did not differ significantly between groups (OR = 0.55, 95% CI: 0.27–1.10, *P* = 0.09).

**Conclusion:**

The available evidence suggests that auriculotherapy is a safe adjunctive therapy for CHF, capable of improving overall clinical effectiveness, enhancing cardiac function, and improving exercise capacity and quality of life.

**Systematic Review Registration:**

PROSPERO CRD42024621500.

## Introduction

Chronic heart failure (CHF) is a complex clinical syndrome characterized by impaired ventricular filling or ejection capacity resulting from structural or functional abnormalities of the heart (1). It represents the severe, terminal stage of various cardiac diseases. The clinical features of CHF vary significantly depending on which cardiac chambers are affected (2). From an epidemiological perspective, heart failure has become a major global public health issue (3). The incidence of CHF continues to increase each year, and mortality rates have risen sharply in recent years, reaching 20% within 1 year and 53% within 5 years (4, 5).

In China, the 2022 “Chinese Guidelines for the Diagnosis and Treatment of Heart Failure” report that heart failure affects 1.3% of adults aged ≥35 years—an estimated 13.7 million people (6). Heart failure is classified according to the affected cardiac chambers as left heart failure, right heart failure, or global heart failure, corresponding to pulmonary congestion, systemic congestion, or a combination of both. Based on left ventricular ejection fraction (LVEF), heart failure can be further classified as heart failure with reduced ejection fraction (HFrEF) (LVEF < 40%, characterized primarily by impaired myocardial contractile function), heart failure with mildly reduced ejection fraction (HFmrEF) (40% ≤ LVEF < 50%, representing an intermediate stage between impaired systolic and diastolic function), or heart failure with preserved ejection fraction (HFpEF) (LVEF ≥ 50%, characterized primarily by impaired myocardial diastolic function). Based on disease onset, heart failure can be classified as acute (sudden worsening of symptoms within a short period, e.g., acute pulmonary edema) or chronic (long-term symptoms that gradually or repeatedly worsen over time) (7). Current heart failure treatment focuses on improving symptoms, delaying progression, and reducing mortality. Existing treatment methods include drug therapy and non-drug therapy, including cardiac resynchronization therapy, heart transplantation, and left ventricular assist devices (8).

Given the limitations of current treatments, auricular therapy, a distinctive method within traditional Chinese medicine (TCM), has increasingly attracted attention. Its theoretical foundation stems from the classical understanding of traditional Chinese medicine, which states that “the ear is closely connected to the internal organs and meridians.” The Ling Shu: Questions on the Mouth states that “The ear is where the meridians converge,” emphasizing that all 12 meridians are directly or indirectly connected to the ear, which forms an integrated connection with the 5 internal organs and 6 viscera through the meridian system. The “inverted fetus” pattern of ear acupuncture point distribution further clarifies the correspondence between the earlobe and the head and face, the ear concha and the thoracic cavity (heart and lungs), and the concha and the abdominal cavity (liver, spleen, and kidneys). By stimulating ear acupuncture points, one can achieve “regulating the flow of qi and blood in the meridians and balancing the yin and yang of the viscera.” For conditions like heart failure, which fall under the categories of “palpitations” and “edema,” ear acupuncture points related to the heart, kidneys, and sympathetic nervous system are often selected to regulate fluid metabolism and cardiac function (9). Modern research reveals the multifaceted mechanisms of ear acupuncture therapy, including inhibiting excessive activation of the sympathetic nervous system via neural reflex pathways, improving heart rate variability to balance autonomic nervous system function, reducing brain natriuretic peptide (BNP) and aldosterone levels to alleviate ventricular remodeling, upregulating vascular endothelial growth factor (VEGF) to improve myocardial microcirculation, and inhibiting the release of pro-inflammatory factors such as TNF-α and IL-6 to enhance the anti-inflammatory capacity of the body and reduce myocardial inflammatory damage (10). Auricular therapy combines traditional acupuncture with modern anatomy to achieve therapeutic effects by stimulating the auricular branch of the vagus nerve (ABVN). It is simple to perform, well-tolerated, safe, cost-effective, and can be used in combination with other interventions (11).

Numerous studies have confirmed its efficacy in improving cardiac function in CHF patients; however, the quality of these studies varies. This study aims to comprehensively analyze the efficacy of auricular therapy in improving cardiac function in CHF patients based on existing clinical trials and to provide a potential supplementary treatment approach for the comprehensive management of heart failure.

## Methods

This meta-analysis and meta-regression analysis was conducted in accordance with the PRISMA and MOOSE guidelines. The protocol was registered beforehand in the International Prospective Register of Systematic Reviews (#CRD42024621500).

### Search strategy

We searched PubMed, Embase, the Cochrane Library, CNKI, Wanfang, VIP, and CBM for publicly available randomized controlled trials (RCTs) on CHF from database inception to 11 November 2025. The search strategy combined subject headings and free-text keywords. The Chinese search terms included auricular acupuncture, auricular acupuncture point, heart failure, and myocardial failure, whereas the English search terms included ear needles, auricular, heart failure, and myocardial failure. A detailed summary of the search strategy is provided in Supplementary Table S3.

### Inclusion and exclusion criteria

The inclusion criteria were as follows: (1) age ≥18 years; (2) patients diagnosed with CHF; (3) the experimental group received auricular therapy, with or without additional treatments; (4) at least one of the following results was recorded: heart function improvement efficiency, LVEF, left ventricular end-diastolic diameter (LVEDD), 6-min walk test, Minnesota Living with Heart Failure Questionnaire (MLHFQ), and adverse reactions; and (5) study type: RCTs.

Exclusion criteria were as follows: (1) other types of articles, such as reviews, letters, conferences, case reports, protocols, meetings, proceedings, abstracts, meta-analyses, etc.; (2) studies with missing or incomplete outcome data; and (3) duplicate publications.

### Study selection

Two reviewers independently screened all records according to the predefined eligibility criteria. They compared their screening decisions, and any disagreements were resolved through discussion or, if necessary, by consulting a third reviewer.

### Data extraction

The data were extracted independently according to the predefined data extraction form, and the extracted information included the following: study inclusion, publication time, sample size, age, disease duration, New York Heart Association (NYHA) cardiac function classification, treatment duration, trial group interventions, acupoints, frequency, and outcome indicators.

### Quality assessment

The risk of bias of the included studies was assessed by two investigators using the Cochrane Collaboration risk of bias assessment tool (12). The two investigators conducted the assessment independently, and any disagreements were resolved through discussion or by consulting a third investigator.

### Data analysis

Statistical analysis was performed using RevMan 5.4. Heterogeneity among studies was assessed via the *p*-value and *I*^2^ statistic: *I*^2^ ≤ 50% with *P* ≥ 0.10 indicated no significant heterogeneity, for which a fixed-effects model was used; *I*^2^ > 50% or *P* < 0.10 suggested significant heterogeneity, for which a random-effects model was adopted, alongside sensitivity analysis to test result stability and subgroup analysis to explore heterogeneity sources. For continuous outcomes, the weighted mean difference (WMD) with a 95% confidence interval (CI) was used when the same measurement tool and unit were consistent across studies. The standardized mean difference (SMD) with 95% CI was applied for outcomes of the same dimension but different measurement tools. Publication bias was assessed using funnel plots.

## Results

### Search results

**Figure 1:**
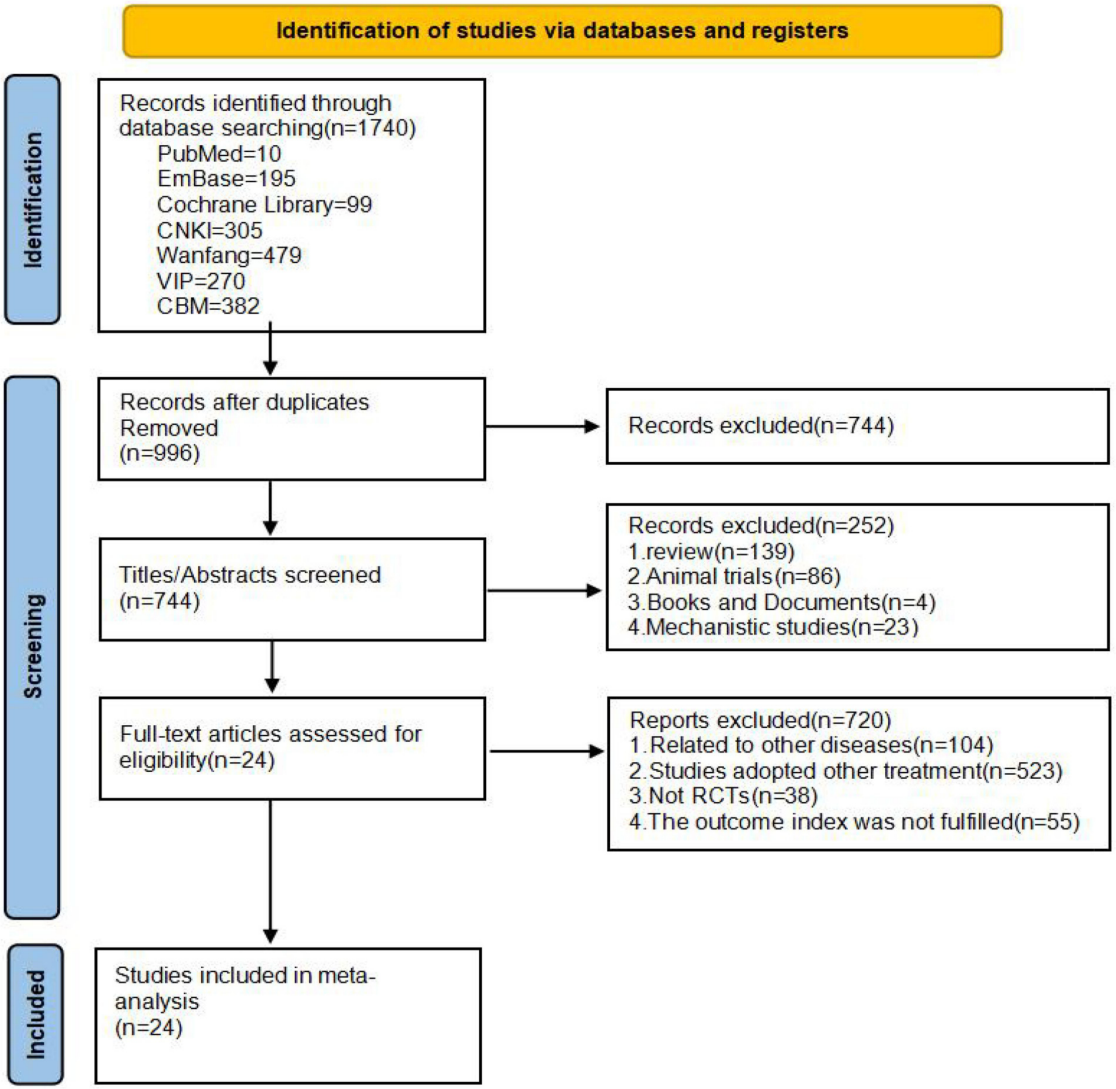
Flowchart of study screening.

A total of 1,740 records were initially retrieved from seven databases. After removal of duplicates, 996 records were retained. Title and abstract screening excluded irrelevant studies, leaving 744 records for full-text assessment. After full-text review, 24 studies met the inclusion criteria and were included in this meta-analysis. The entire screening process is shown in Figure 1.

### Characteristics of included studies

A total of 24 RCTs were included, involving 2,387 study participants—1,195 in the observation group and 1,192 in the control group. The basic characteristics of the included studies are presented in Table 1.

**Table 1:** Basic characteristics of included studies.

Study	Year	Sample size (T/C)	Age (T/C)	Duration (T/C)	NYHA	Duration	Intervention	Acupoints	Frequency	Outcome
Yan (13)	2021	43/43	61.2 ± 3.8/61.6 ± 3.7	NR	2–4	4 weeks	Magnetic bead pressure on auricular points combined with calisthenics training + conventional treatment	Heart, kidney, lung, sympathetic nervous system, Shenmen	3–5 times per day, 1–3 min of acupoint pressing each time; auricular patches replaced every 2–7 days, with a 7-day course of treatment (total four courses); alternate between two ears	②③④⑤
Tian (14)	2023	52/52	71.50 ± 7.55/70.83 ± 7.36	24, 11, 17/18, 17, 17	2–3	3 weeks^a^	Auricular snap-needle + conventional treatment	Heart, kidney, subcortex, ear center, Shenmen, small intestine	3–5 times per day, no less than 30 s per acupoint; the needle is retained for 3 days after embedding, with a 1-day interval before re-embedding; acupoints on one ear are selected for each embedding, and operations alternate between two ears	①④⑤
Liu (15)	2023	40/40	74.23 ± 7.27/71.38 ± 12.42	NR	NR	10d	Auricular acupressure combined with hanging moxibustion treatment + conventional treatment	Heart, sympathetic nervous system, kidney, Shenmen, endocrine, spleen	3 times per day, 2 min each time; alternate between two ears	①⑤
Li (16)	2024	40/40	72.51 ± 7.71/72.52 ± 7.81	NR	NR	NR	Auricular pressure bean + conventional treatment	Subcortex, Shenmen, small intestine, kidney, bladder	3 times per day, 2–3 min each time; Vicia seed and adhesive tape replaced every 3 days	②
Tang (17)	2021	120/120	66.11 ± 12.07/68.84 ± 9.85	7.18 ± 8.22/5.53 ± 5.4	2–3	2 weeks	Auricular pressure bean combined with extracorporeal counterpulsation therapy + conventional therapy	Heart, lung, spleen, kidney, endocrine, Shenmen, sympathetic nervous system	3–5 times per day, 3–5 min per acupoint each time; each ear is pressed for 3 days, continuous treatment for 2 weeks	①⑤
Zhang (18)	2022	45/45	59.15 ± 6.21/58.39 ± 6.17	NR	2–3	12 days	Auricular pressure bean + conventional treatment	Heart, lung, sympathetic nervous system, adrenal gland, trachea, throat, endocrine	3 times per day, 2 min each time; auricular patches replaced every 3 days, switching to the opposite ear; continuous treatment for 12 days	①⑥
Liu (19)	2024	40/40	53.29 ± 3.15/53.19 ± 3.14	2.64 ± 0.22/2.59 ± 0.22	2–3	2 months	Auricular pressure bean combined with a herbal footbath + conventional treatment	Heart, kidney, Pishu	3 times per day: knead for 20 s, rest for 10 s, then continue kneading (three rounds per acupoint); Vicia seed replaced every 3 days; alternate between two ears	④
Liu (20)	2020	43/43	66.25 ± 5.23/65.72 ± 5.14	NR	NR	36 days	Auricular acupressure + conventional treatment	Kuaihuo, Shenxin, Shenmen, subcortex, liver, heart	3 times per day, 3–5 min each time; switch to the opposite ear every 3 days, alternate pressing between two ears; continuous intervention for 36 days	⑤
Zhao (21)	2023	48/48	72.66 ± 1.31/72.68 ± 1.28	5.24 ± 0.29/5.21 ± 0.36	2–3	2 weeks	Auricular pressure bean combined with Wen Yang Yi Xin Tang + conventional treatment	Auricular heart, auricular sympathetic nervous system, auricular kidney, auricular subcortex	Once every other day, 0.5–1 min per acupoint (3 times per acupoint); continuous treatment for two courses (1 course = 7 days)	①②④⑥
Qin (22)	2024	44/44	67.44 ± 6.34/67.61 ± 6.25	12.22 ± 4.76/12.31 ± 4.59	2–4	1 month	Auricular seed embedding combined with warm acupuncture on the dorsal Yu points + conventional treatment	Heart, liver, kidney, Shenmen, endocrine, subcortex, sympathetic nervous system	3–4 times per day, 2 min each time; embedded seeds replaced every 3 days; continuous intervention for 1 month	①②③
Dai CW (23)	2022	30/30	73.12 ± 6.1/73.28 ± 6.42	NR	NR	1 month	Auricular pressure bean combined with acupressure + conventional treatment	Sympathetic nervous system, Shenmen, heart, subcortex, heart point, chest	Once per day; Vicia seed replaced every 2 days; total treatment duration of 1 month	①②③
Wu (24)	2024	48/47	63.21 ± 6.95/65.51 ± 7.35	6.08 ± 1.05/5.88 ± 1.25	2–3	6 weeks	Guan's auricular acupuncture combined with nourishing heart soup plus reduction + conventional treatment	NR	Once per day; acupuncture performed alternately on acupoints of the two ears every other day; treat for 6 days, rest for 1 day; total treatment for 6 weeks	①②③④
Liu (25)	2023	100/100	68.72 ± 10.02/72.37 ± 10.06	NR	2–4	4 weeks	Auricular pressure pills combined with the formula of benefiting qi and activating blood, warming yang and inducing diuresis + conventional treatment	Heart, kidney, sympathetic nervous system, Shenmen, subcortex	3–5 times per day, 1–2 min each time; replaced every other day; treatment for 4 weeks	①②⑤
Fan (26)	2016	34/32	68.62 ± 6.21/67.87 ± 6.44	3.01 ± 1.81/2.88 ± 1.93	2–4	4 weeks	Auricular acupressure combined with Yixin Tang + conventional treatment	NR	3 times per day, 1–2 min per acupoint each time; alternate between two ears, replaced every other day; 4-week course of treatment	①②
Wang (27)	2021	30/30	54.27 ± 4.72/55.21 ± 5.12	2.61 ± 0.53/2.35 ± 0.61	2–3	2 weeks	Auricular seed embedding combined with acupoint application + conventional treatment	Heart, kidney, endocrine, spleen, Sanyinjiao (SP6)	3–5 times per day, 1–2 min per acupoint each time; alternate use of bilateral auricular acupoints	①
Wang (28)	2023	54/54	72.51 ± 9.67/75.74 ± 8.81	1.58 ± 1.28/1.58 ± 1.33	NR	1 month	Auricular pressure bean combined with TCM acupressure + conventional treatment	Sympathetic nervous system, Shenmen, heart, cortex	Once per day, alternate pressing; continuous treatment for 1 month	①②⑥
Wu (29)	2023	39/38	66.51 ± 8.73/64.25 ± 8.51	6.81 ± 2.52/6.44 ± 2.23	NR	6 weeks	Auricular pressure bean + conventional treatment	Heart, lung, kidney, Shenmen	3 times per day, 1–2 min per acupoint each time; replaced every other day, pressing acupoints on the opposite ear; 2-week course of treatment (total three courses)	①②④
Geng (30)	2025	30/30	66.03 ± 6.60/67.30 ± 6.08	NR	2–3	4 weeks	Auricular pressure bean + conventional treatment	Heart, lung, spleen, kidney, adrenal gland, sympathetic nervous system, subcortex, endocrine	3 times per day, 2 min each time; auricular patches replaced every other day, switching to the opposite ear; continuous treatment for 4 weeks	②④
Lin (31)	2024	104/104	61.52 ± 1.75/61.41 ± 1.82	3.98 ± 1.15/3.99 ± 1.12	2–3	10 days	Moxibustion, acupoint application, auricular seed pressure + conventional treatment	Heart, lung, spleen, liver, kidney, Shenmen, endocrine	Directly apply Vicia seed patches to acupoints and knead appropriately; replace with disinfection every other day	④
Tang (32)	2024	60/60	68.63 ± 5.14/69.01 ± 5.21	10.45 ± 2.05/10.21 ± 2.13	2–3	8 weeks	Qiti Yixin decoction combined with auricular needle embedding + conventional treatment	Shenmen, sympathetic nervous system, subcortex, heart, liver, ear center	Once per day, 3 min per acupoint; replaced every 3 days; alternate use of two ears	①②③④
Zhu (33)	2024	35/35	71.62 ± 4.33/71.54 ± 4.37	NR	2–4	1 months	Acupoint application combined with auricular seed pressing + conventional treatment	Endocrine acupoint, stomach acupoint, liver acupoint, sympathetic nervous system, auricular acupoint, auricular Shenmen acupoint	3 times per day, 3 min per acupoint; Vicia seed replaced every 2 days	④
Fang (34)	2025	45/45	62.52 ± 6.81/61.91 ± 7.25	NR	2–4	4 weeks	TCM foot bath combined with auricular seed pressing + conventional treatment	Heart, lung, spleen, kidney, Shenmen, sympathetic nervous system	3–5 times per day, about 30 s per acupoint; alternate between two ears; replaced every 3 days	②③⑥
Lu (35)	2025	40/40	74.23 ± 4.76/73.89 ± 3.92	4.98 ± 1.27/5.07 ± 1.43	2–4	12 weeks	Qiti Yixin decoction combined with auricular needle embedding + conventional treatment	Auricular heart, auricular lung, auricular kidney, auricular Shenmen, and auricular sympathetic nervous system	3–5 times per day, 1–2 min per acupoint each time; thumbtack needles replaced every 3 days; continuous treatment for 12 weeks	②
Huang (36)	2024	39/40	69.15 ± 8.08/69.83 ± 6.92	6.99 ± 2.83/7.76 ± 3.34	2–3	4 weeks	Moxibustion combined with acupoint application, auricular seed pressing + conventional treatment	Heart, kidney, Shenmen, sympathetic nervous system, subcortex	4–5 times per day, 3–5 min each time; retained for 1 day	②④⑤⑥

T, test group; C, control group; NR: not reported; outcome indicators: ① cardiac function improvement efficiency, ② left ventricular ejection fraction, ③ left ventricular end-diastolic internal diameter, ④ 6MWT, ⑤ Minnesota Quality of Life Score, ⑥ adverse effects; the interventions in the control group were all conventional treatments.

a Is divided according to the duration of the disease ≤1, 2–5, ≥6.

### Risk of bias assessment

**Figure 2:**
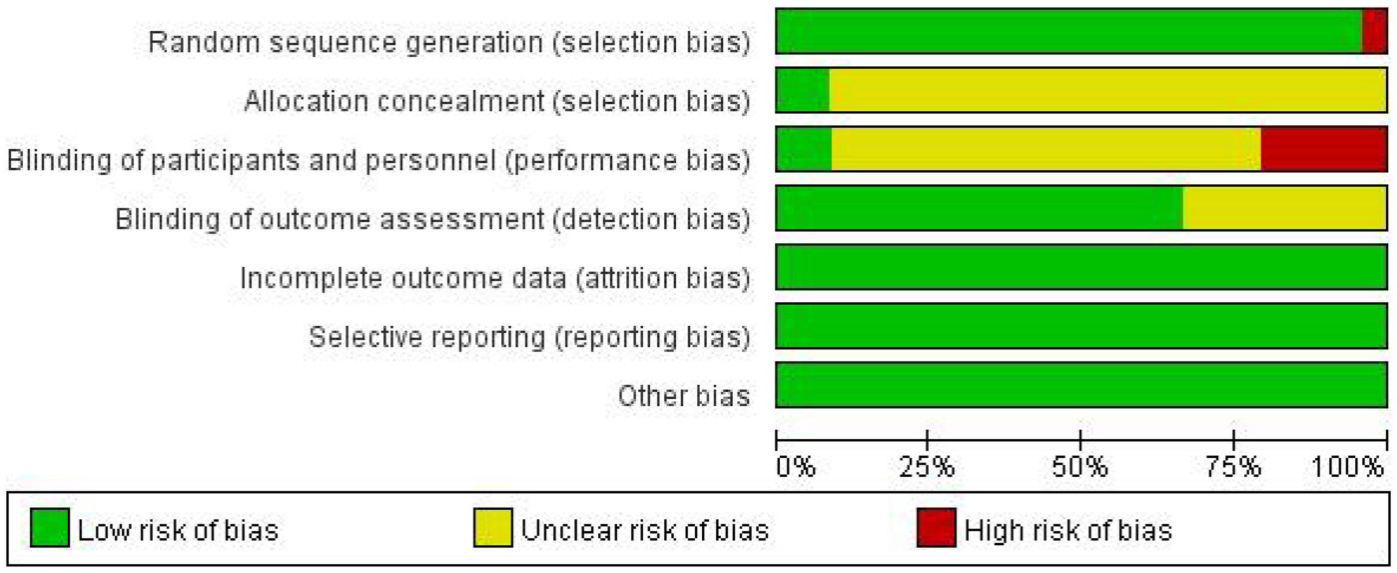
Risk of bias assessment of the included studies.

The Cochrane Collaboration's tool for assessing risk of bias was used to evaluate the quality of the included studies. The evaluation results are shown in Figure 2.

### Meta-analysis results

#### Total effective rate

**Figure 3:**
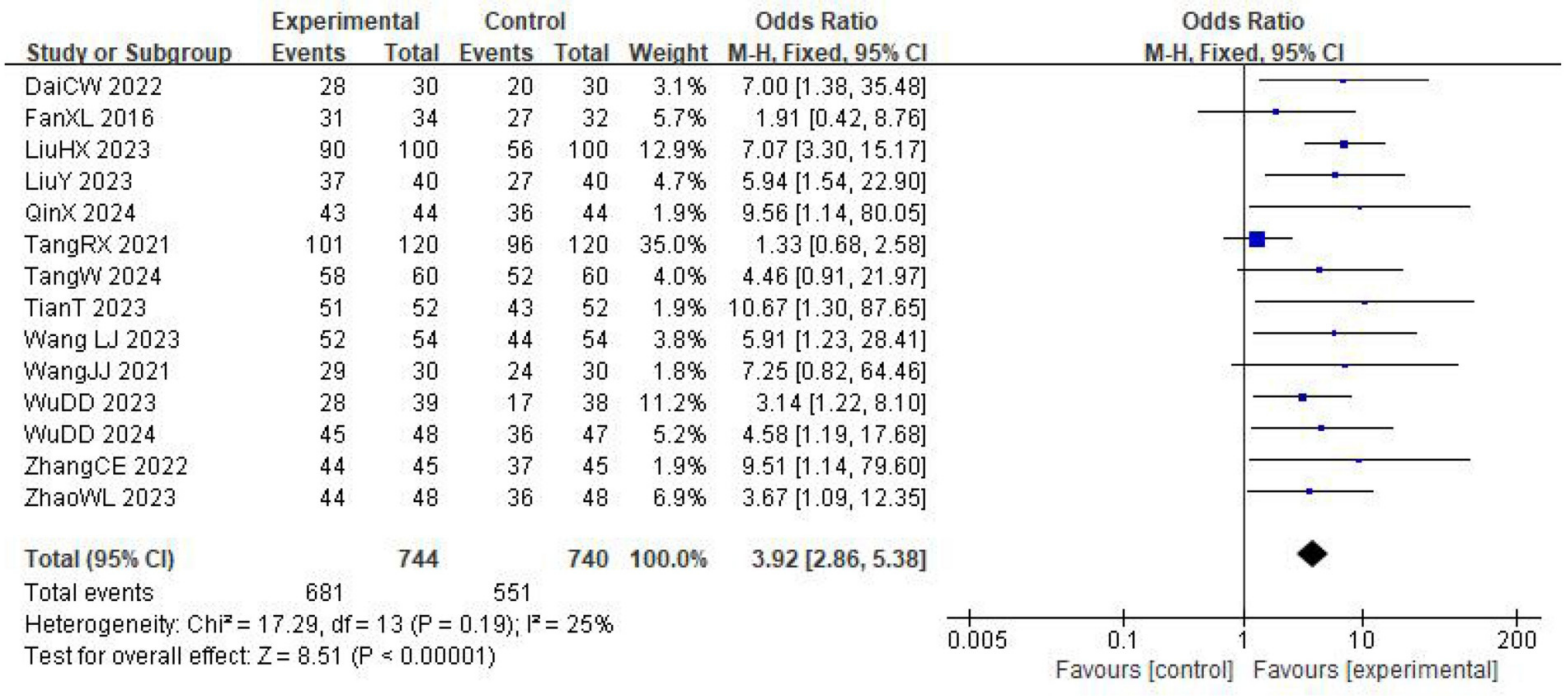
Meta-analysis of the effective rate of auricular acupoint therapy on the improvement of cardiac function.

Fourteen trials involving 1,484 patients (744 in the experimental group and 740 in the control group) reported the clinical effective rate for cardiac function improvement (Figure 3). The heterogeneity test indicated low between-study heterogeneity (*I*^2^ = 25%, *P* = 0.19), so a fixed-effects model was employed for the meta-analysis. The results revealed that the clinical effective rate for cardiac function improvement in the experimental group was significantly higher than in the control group (OR = 3.92, 95% CI: 2.86–5.38, *P* < 0.00001). Subgroup analyses based on treatment duration, sample size, and intervention measures did not identify any significant sources of heterogeneity. Sensitivity analyses were conducted by sequentially excluding individual studies. Removing the study by Tang et al. study resulted in no significant heterogeneity among the studies (*I*^2^ = 0%, *P* = 0.95). A fixed-effects model was then used for the meta-analysis. The results showed that the cardiac function improvement rate in the experimental group remained significantly higher than in the control group (OR = 5.32, 95% CI: 3.68–7.71, *P* < 0.00001).

The main difference between the trial by Tang et al. and the other included studies lies in the composition of the combined intervention. Tang et al. used external counterpulsation in combination with auricular point pressing using Vaccaria seeds, whereas the other trials combined auricular point pressing mainly with non-device-based therapies such as herbal decoctions, moxibustion, or structured exercise programs. External counterpulsation is a mechanical, device-based therapy that improves systemic circulation and enhances coronary perfusion. Its mechanism of action differs fundamentally from the pharmacological effects of herbal decoctions, the thermal stimulation of moxibustion, and the functional rehabilitation achieved through exercise training. It may also act synergistically with auricular point pressing, leading to larger improvements in cardiac function than those observed in trials without external counterpulsation. This mechanistic divergence is likely to contribute substantially to the between-study heterogeneity.

#### Left ventricular ejection fraction

**Figure 4:**
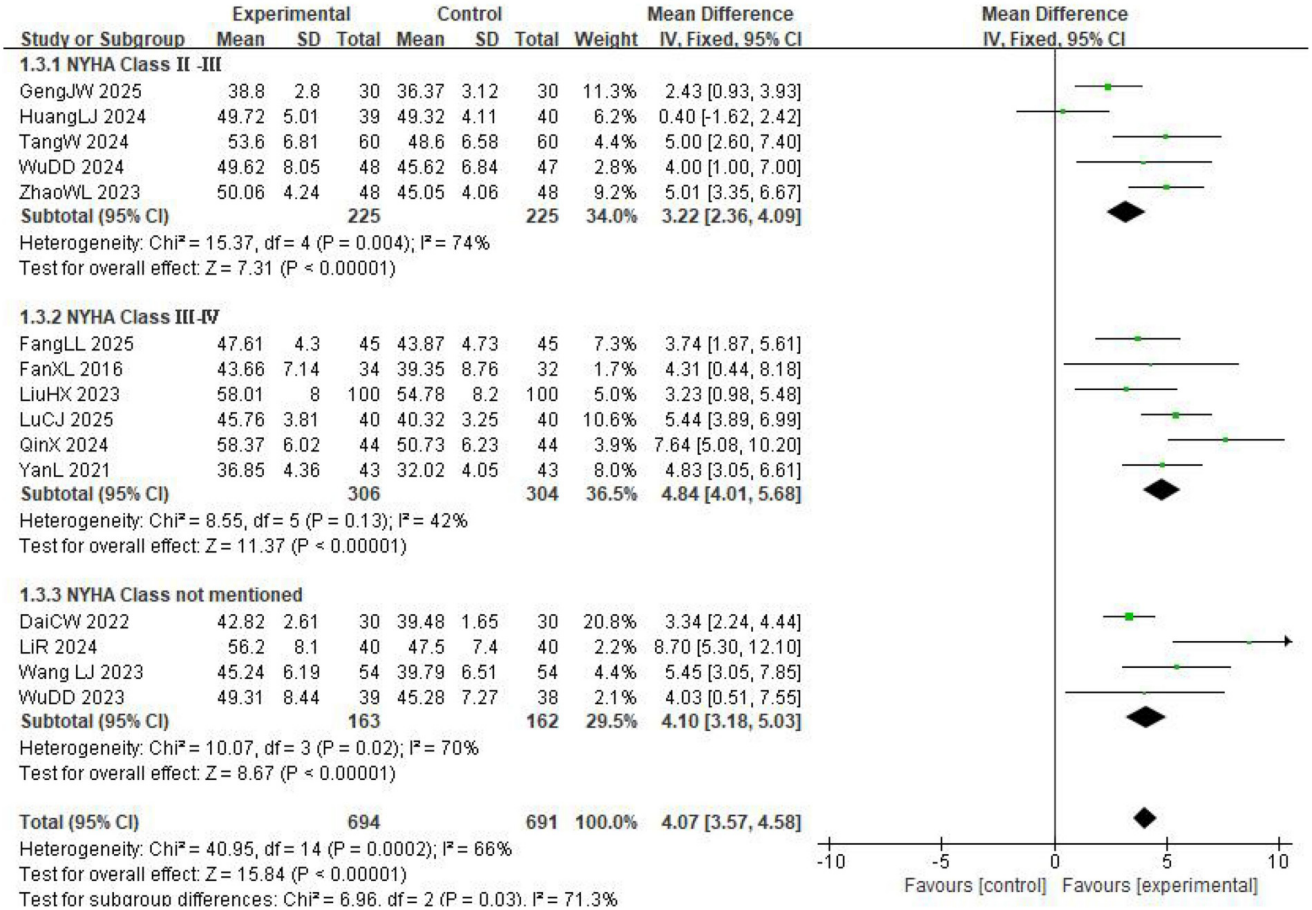
Meta-analysis of auricular acupoint therapy on LVEF in patients.

Fifteen studies reported changes in LVEF (Figure 4), and the pooled results indicated substantial between-study heterogeneity (*I*^2^ = 66%, *P* = 0.0002). Therefore, a random-effects model was used for the meta-analysis. The results revealed that LVEF in the experimental group was significantly higher than in the control group, suggesting that the experimental group achieved greater effectiveness in improving LVEF in patients with CHF (MD = 4.33, 95% CI: 3.41–5.25, *P* < 0.00001). To explore the sources of heterogeneity in LVEF, a subgroup analysis was conducted based on the NYHA class. The subgroup of patients with NYHA class II–III included five studies and exhibited significant heterogeneity (*I*^2^ = 74%, *P* = 0.004); however, the results still indicated that the experimental group achieved a greater improvement in LVEF than the control group (MD = 3.22, 95% CI: 2.36–4.09, *P* < 0.00001). The subgroup of patients with NYHA class III–IV comprised six studies, with no substantial heterogeneity among the studies (*I*^2^ = 42%, *P* = 0.13), and the analysis demonstrated that the experimental group had a superior effect on LVEF improvement compared with the control group (MD = 4.84, 95% CI: 4.01–5.68, *P* < 0.00001). The subgroup of studies that did not report NYHA class included four trials and showed significant heterogeneity (*I*^2^ = 70%, *P* = 0.02), but the results consistently revealed that the experimental group had a more pronounced improvement in LVEF than the control group (MD = 4.07, 95% CI: 3.57–4.58, *P* < 0.00001).

Overall, the subgroup analyses showed that the experimental intervention exerted a broadly consistent beneficial effect on LVEF across patients with different baseline cardiac function statuses and was not significantly constrained by cardiac function classification. The absence of substantial heterogeneity in the NYHA class III–IV subgroup may be attributed to patients’ more severe disease, as well as greater standardization of their pathophysiological status and background treatments, which enhanced the consistency of intervention effects across studies.

#### Left ventricular end-diastolic diameter

Six studies reported changes in LVEDD (Supplementary Figure S1). Substantial heterogeneity was identified (*I*^2^ = 83%, *P* < 0.00001), so a random-effects model was applied. Subgroup analyses based on treatment duration, sample size, and experimental intervention did not identify clear sources of heterogeneity. To further explore the source of heterogeneity related to LVEDD, a subgroup analysis was performed according to cardiac function classification. The subgroup of patients with NYHA class II–III included two studies and exhibited significant heterogeneity (*I*^2^ = 95%, *P* < 0.00001); however, the analysis still showed that the experimental group achieved a greater reduction in LVEDD than the control group (MD = −4.76, 95% CI: −6.13 to −3.38, *P* < 0.00001). The subgroup of patients with NYHA class II–IV comprised three studies, with no substantial heterogeneity among the studies (*I*^2^ = 0%, *P* = 0.74), and the results indicated that the experimental group achieved a greater improvement in LVEDD compared with the control group (MD = −3.31, 95% CI: −4.35 to −2.28, *P* < 0.00001). One study did not specify the cardiac function classification and was therefore excluded from the subgroup meta-analysis.

No significant heterogeneity was seen in the NYHA class II–IV subgroup, which is likely related to patients’ more advanced disease, more uniform patterns of left ventricular remodeling, and more standardized background treatments, all of which enhance the consistency of intervention effects across studies. In contrast, significant heterogeneity existed in the NYHA class II–III subgroup, possibly due to milder disease status, larger baseline variations in LVEDD, and inconsistencies in intervention details or background treatments across studies.

#### Six-minute walking distance

Eleven studies reported the 6-min walk distance (6MWD) (Supplementary Figure S2), and the heterogeneity test showed a high degree of between-study heterogeneity (*I*^2^ = 96%, *P* < 0.00001). Therefore, a random-effects model was used for the meta-analysis. To explore the sources of this heterogeneity, a subgroup analysis was performed based on acupoint combinations, yielding the following results. The cardiopulmonary–kidney (CPK)-based variable-acupoint subgroup included five studies and exhibited significant heterogeneity among studies (*I*^2^ = 96%, *P* < 0.00001). Nevertheless, the results indicated that the experimental group exhibited greater improvement in the 6MWD than the control group (MD = 71.06, 95% CI: 68.27–73.85, *P* < 0.00001). The non-CPK-based variable-acupoint subgroup included six studies, with no substantial heterogeneity among studies (*I*^2^ = 0%, *P* = 0.48), and the analysis showed that the experimental group also had a better improvement in 6MWD than the control group (MD = 32.49, 95% CI: 25.95–39.03, *P* < 0.00001).

Although the CPK-based variable-acupoint subgroup used fixed core acupoints, it still exhibited marked heterogeneity. This may be attributable to differences in the types of auxiliary acupoints used, as well as variations in the ratio of core to auxiliary acupoints, stimulation methods, and treatment duration, which together altered intervention targets and synergistic effects. In contrast, the non-CPK-based variable-acupoint subgroup utilized non-core acupoints with relatively fixed combinations; as a result, no significant heterogeneity was observed.

#### MLHFQ scores

Seven studies examined the impact of auricular therapy on the quality of life of patients with CHF (Supplementary Figure S3). The results revealed significant heterogeneity (*I*^2^ = 88%, *P* < 0.0001), prompting the use of a random-effects model for analysis. The results indicated that MLHFQ scores were lower in the experimental group than those in the control group after the intervention, with statistically significant differences (MD = −7.29, 95% CI: −8.52 to −6.06, *P* < 0.00001). To investigate the sources of heterogeneity in the MLHFQ results, subgroup analysis was performed based on the mean age of patients. For patients with a mean age <65 years, only one study was included; therefore, this subgroup was not incorporated into the meta-analysis. For patients with a mean age of 65–70 years, four studies were included, with no significant heterogeneity among studies (*I*^2^ = 0%, *P* = 0.66), indicating that the improvement in MLHFQ scores in the experimental group was superior to that in the control group (MD = −4.00, 95% CI: −5.55 to −2.45, *P* < 0.00001). For patients with a mean age > 70 years, two studies were included, with no significant heterogeneity among studies (*I*^2^ = 0%, *P* = 0.49), suggesting that the experimental group in this subgroup also achieved a greater improvement in MLHFQ scores than the control group (MD = −10.51, 95% CI: −13.66 to −7.36, *P* < 0.00001).

Based on the subgroup analysis, the heterogeneity among studies may be attributed to differences in the mean age of patients. In summary, no heterogeneity was observed within either age-based subgroup, primarily because age stratification helped homogenize the physiological status and treatment regimens of patients within each subgroup, thereby reducing variability. Among patients with CHF, those with a mean age >70 years exhibited more pronounced improvements in quality of life after receiving auricular therapy. This phenomenon may be associated with more severe baseline clinical symptoms in this age group, leading to a greater marginal improvement effect following intervention. In addition, it may be related to more standardized chronic disease management and higher treatment adherence among older patients.

#### Adverse reactions

Five studies reported adverse effects. The heterogeneity test suggested no substantial heterogeneity (*I*^2^ = 0%, *P* = 0.46); therefore, a meta-analysis was performed using a fixed-effects model. The results showed that there was no statistically significant difference in the incidence of adverse reactions between the two groups (OR = 0.55, 95% CI: 0.27–1.10, *P* = 0.09).

These findings suggest that auricular therapy has a safety profile comparable to that of conventional treatment and does not substantially increase the risk of adverse events in patients with CHF. This is likely related to the minimally invasive nature and inherently low procedural risk of auricular therapy. Moreover, control groups generally received standard medical therapy, resulting in similar baseline safety risks between treatment arms. The relatively consistent monitoring of adverse events, intervention procedures, and baseline patient characteristics across trials may also have reduced variability in safety outcomes. Overall, the evidence supports the clinical use of auricular therapy as a safe adjunctive treatment method for CHF.

#### Publication bias and sensitivity analysis

For outcome indicators with more than 10 included studies, funnel plots were used to assess publication bias. All funnel plots of the three outcome indicators in this study showed a significant asymmetric distribution (Supplementary Figure S4). Heterogeneity analysis revealed that moderate to high heterogeneity existed among all three indicators, which might be attributed to differences in intervention details, baseline characteristics of the study population, and intervention duration across the included studies. These factors led to the dispersion of effect sizes, thereby affecting the symmetry of the funnel plots. Sensitivity analysis adopted the strategy of sequentially excluding individual studies. By removing each study one by one and recalculating the combined effect size, the impact of a single study on the overall combined results was evaluated. The results revealed that after excluding any single study, neither the direction nor the magnitude of the combined effect size changed significantly, demonstrating that the quantitative combined results of this study had good stability and high reliability.

## Discussion

This meta-analysis, including 2,387 patients with chronic heart failure, demonstrated that auricular therapy combined with standard care significantly improved left ventricular ejection fraction, reduced left ventricular end-diastolic diameter, and increased 6-min walk distance, thereby contributing to meaningful improvements in overall cardiac function (37, 38). The combination therapy also enhanced the quality of life of patients, consistent with the findings reported by Jiang et al. (39). Importantly, the addition of auricular therapy did not increase the incidence of adverse events, indicating that it is a safe and potentially valuable adjunctive intervention in the comprehensive management of chronic heart failure.

Recent advances in auricular vagus nerve stimulation (aVNS) research have provided a clearer mechanistic rationale for the clinical benefits observed in this study. Functional neuroimaging and physiological studies have demonstrated that commonly used auricular points, such as the heart, sympathetic nervous system, subcortex, and Shenmen points, are densely innervated by the ABVN. Stimulation of these sites activates the ABVN–nucleus tractus solitarius (NTS) pathway, enhances vagal efferent activity, suppresses sympathetic nervous system overactivation, and improves heart rate variability and baroreflex sensitivity (40). This autonomic rebalancing aligns well with the characteristic “vagal withdrawal–sympathetic dominance” observed in heart failure and may help reduce myocardial oxygen consumption and improve ventricular systolic–diastolic coupling, thereby supporting the improvements in left ventricular function found in the present analysis. Experimental and clinical evidence further indicates that transcutaneous aVNS (taVNS) can ameliorate ventricular remodeling, reduce myocardial fibrosis, and enhance cardiac performance, effects that are closely associated with autonomic restructuring (41). These findings suggest that auricular stimulation may exert both short-term regulatory effects on cardiac autonomic function and long-term modulatory effects on pathological myocardial remodeling. In addition to autonomic modulation, emerging neuroimmune research highlights the vagus nerve as a key regulator of inflammatory pathways. taVNS has been shown to suppress pro-inflammatory cytokines such as TNF-α and IL-1β (42), thereby mitigating the chronic low-grade inflammation commonly observed in heart failure. Reduction of systemic inflammation may improve skeletal muscle metabolic efficiency and exercise tolerance, which aligns with the observed improvements in 6-min walk distance and patient-reported quality of life in this study. Importantly, recent systematic reviews demonstrate that taVNS is generally well tolerated, with a low incidence of adverse events (43), consistent with the safety profile observed in the included trials.

All included studies were published in Chinese journals, reflecting the predominance of auricular therapy within traditional Chinese medicine and integrative cardiovascular care settings. As a distinctive branch of TCM, auriculotherapy is theoretically rooted in traditional Chinese medical theories and remains relatively niche in international medical research. Currently, most researchers in this field—especially those focusing on its application in chronic heart failure—are from China. Relying on China's abundant TCM clinical resources, these domestic scholars have conducted extensive practical studies, and their findings are mostly published in Chinese. Internationally, the understanding and acceptance of TCM theories and auriculotherapy are limited, resulting in low enthusiasm among foreign scholars to engage in relevant research and a scarcity of English-language study outputs. Despite multichannel searches during the study, English-language studies meeting the inclusion criteria were still extremely scarce. Thus, only Chinese-language studies were included in this study.

Eleven studies enrolled participants with heart function grades of 2–3, seven studies enrolled participants with grades of 2–4, and six studies did not specify the grading system used. Fifteen studies employed Vaccariae Semen auricular point pressing, three studies used magnetic bead acupoint application for stimulation, two studies employed medicinal bean acupoint pressing, and an additional four studies utilized filiform needles combined with tube-type intradermal needles as the intervention method. Acupoints were identified by probing sensitive areas with an ear probe. The area was then disinfected with 75% ethanol, and the selected stimulus was applied. The intensity of stimulation was adjusted until the patient experienced a sensation of acidity or pain. Although the overall procedure was similar, standardized operating procedures were not established for specific aspects, such as acupoints, frequency, and duration of treatment. Despite this variability, the overall findings consistently indicated multidimensional benefits of auricular therapy across cardiac function, exercise capacity, and quality of life—outcomes that likely reflect the convergence of autonomic modulation, cardiac reflex pathway regulation, and neuroimmune mechanisms. All included studies reported that auricular therapy was effective in treating CHF (*P* < 0.05).

Nevertheless, several limitations should be acknowledged. First, the methodological quality of the included studies was suboptimal, with insufficient reporting of randomization, allocation concealment, and blinding, which may introduce bias. Second, auricular therapy protocols lacked standardization, resulting in heterogeneity in point selection, stimulation modalities, and treatment durations. Third, most studies did not incorporate sham auricular controls, making it difficult to distinguish specific effects from placebo responses. In addition, all included studies were conducted in China, which may limit the generalizability of results due to the absence of international multicenter trials.

Future research should focus on developing standardized auricular therapy protocols, establishing core point prescriptions, optimizing stimulation parameters, and recommending treatment durations. Rigorous randomized, double-blind, sham-controlled multicenter trials are needed to strengthen the evidence base. Incorporating objective physiological indicators, such as heart rate variability, ambulatory ECG monitoring, and inflammatory biomarkers, may also help elucidate the mechanistic pathways. Furthermore, increased publication of high-quality English-language studies will facilitate broader academic recognition and support the integration of auricular therapy into global heart failure management strategies.

## Data Availability

The original contributions presented in the study are included in the article/Supplementary Material; further inquiries can be directed to the corresponding author.
