# Supplementary material for: Auriculotherapy for the intervention effect of chronic heart failure: a systematic review and meta-analysis
Source: Front Cardiovasc Med. 2026 Jan 8;12:1685507. doi: 10.3389/fcvm.2025.1685507 (PMC12823894; doi:10.3389/fcvm.2025.1685507)

Supplementary Table 1: PRISMA Checklist

| **Section/topic** | **#** | **Checklist item** | **Reported on page #** |
| --- | --- | --- | --- |
| **TITLE** | | |  |
| Title | 1 | Auriculotherapy for the intervention effect of chronic heart failure: A systematic review and meta-analysis | 1 |
| ABSTRACT | | |  |
| Structured summary | 2 | Structured summary including: background; objectives; data sources; study eligibility criteria, participants, and interventions; study appraisal and synthesis methods; results; limitations; conclusions and implications of key findings. | 1 |
| INTRODUCTION | | |  |
| Rationale | 3 | Rationale for the review in the context of current knowledge (chronic heart failure burden and limited efficacy of existing treatments, potential of auriculotherapy). | 1 |
| Objectives | 4 | To systematically evaluate the efficacy and safety of auriculotherapy in chronic heart failure (CHF). Reported in the objectives section of introduction. | 2 |
| METHODS | | |  |
| Protocol and registration | 5 | Protocol registered in the International Prospective Register of Systematic Reviews (#CRD42024621500). | 2 |
| Eligibility criteria | 6 | Inclusion and exclusion criteria (RCTs on auriculotherapy for CHF; excluding non-RCTs, duplicate publications, etc.) are reported in the methods section. | 3 |
| Information sources | 7 | PubMed, EMbase, Cochrane Library, CNKI, Wanfang, VIP, CBM databases, searched up to November 11, 2025. No contact with authors for additional information. | 3 |
| Search | 8 | Electronic search strategy using combined subject and free words: Chinese terms (auricular acupuncture, heart failure, etc.); English terms (Ear needles, Auricular, heart failure, etc.). | 3 |
| Study selection | 9 | Process for selecting studies is described in PRISMA flow chart (Figure 1), with two reviewers independently screening and resolving disputes via discussion or third-party adjudication. | Figure 1 |
| Data collection process | 10 | Author 1 and Author 2 independently evaluated the data for quality. Inconsistencies were resolved on the assessment by the corresponding author. | 3 |
| Data items | 11 | Outcomes of interest for data collection are reported in the manuscript. | 3 |
| Risk of bias in individual studies | 12 | Risk of bias in individual studies assessed using the Cochrane Collaboration risk of bias assessment tool. | Figure 2 |
| Summary measures | 13 | Relative risk (RR) for effective rate and adverse reactions; mean differences (MD) for LVEF, LVEDD, 6MWT; standardized mean differences (SMD) for MLHFQ. | 3 |
| Synthesis of results | 14 | The fixed effect (Mantel–Haenszel) model was used when there was no significant heterogeneity and the random effects (Inverse variance) model was used for those with significant heterogeneity. Heterogeneity was defined as low, moderate, and high based on I square value (<40%: low; 30%–60%: moderate; 50%–90%: substantial >75%: high). Heterogeneity with a high I square value >50% was considered statistically significant. | 3 |
| Risk of bias across studies | 15 | Publication bias assessed by visual analysis of funnel plot. | 3 |
| Additional analyses | 16 | Leave-one out sensitivity analysis for outcomes with statistically significant heterogeneity. | 3 |
| RESULTS | | |  |
| Study selection | 17 | Explained in PRISMA flow diagram. | Figure 1 |
| Study characteristics | 18 | Characteristics of the included studies are described in Table 1. | Table 1 |
| Synthesis of results | 19 | Results of meta-analysis for each outcome are reported in results section and Figures. | Figure 3, 4 and Supplementary Figure 1,2,3 |
| Risk of bias across studies | 20 | Funnel plot was used to evaluate the publication bias across studies. | Supplementary Figure 4 |
|  |  |  |  |
| Summary of evidence | 21 | Summary of main findings (auriculotherapy improves efficacy and safety in CHF) and relevance reported in discussion section. | 17-18 |
| Limitations | 22 | Limitations of the study (retrospective design, heterogeneity in intervention protocols, limited high-quality RCTs) reported in discussion. | 18 |
| Conclusions | 23 | Auriculotherapy can significantly improve clinical efficacy and cardiac function in CHF with good safety; clinical application requires standardized protocols. | 18 |
|  | 24 |  |  |
| Funding | 25 | This study received no funding. | 19 |

Supplementary Table 2: MOOSE Checklist

| Item No | Recommendation | Brief description of how the criteria were satisfied |
| --- | --- | --- |
| Reporting of background should include | | |
| 1 | Problem definition | Review of literature revealed that auriculotherapy is a complementary therapy, but its clinical application in chronic heart failure (CHF) is limited due to the absence of standardized efficacy evaluation indicators, which is referenced in the introduction. |
| 2 | Hypothesis statement | To systematically evaluate the efficacy and safety of auriculotherapy in CHF. This is reported in the objectives section of the introduction. |
| 3 | Description of study outcome(s) | Meta-analysis of outcomes including cardiac function improvement efficiency, left ventricular ejection fraction (LVEF), left ventricular end-diastolic diameter (LVEDD), 6-minute walk test (6MWT), Minnesota Living with Heart Failure Questionnaire (MLHFQ) scores, and adverse reactions. |
| 4 | Type of exposure or intervention used | Efficacy and safety of auriculotherapy (alone or combined with other methods) in treating CHF, compared with conventional treatment. |
| 5 | Type of study designs used | Randomized controlled trials (RCTs). |
| 6 | Study population | Patients with CHF meeting recognized or authoritative diagnostic criteria, regardless of race, nationality, age, or disease duration. |
| Reporting of search strategy should include | | |
| 7 | Qualifications of searchers | Credentials of authors listed in the author list. |
| 8 | Search strategy, including time period and key words | Databases were searched from inception to November 11, 2025. Chinese search terms: auricular acupuncture, auricular acupuncture point, heart failure, myocardial failure, etc. English search terms: Ear needles, Auricular, heart failure, Myocardial Failure, etc. |
| 9 | Effort to include all available studies | All RCTs meeting inclusion criteria were included, and data from these studies was sufficient for meta-analysis without needing to contact authors. |
| 10 | Databases and registries searched | PubMed, EMbase, Cochrane Library, CNKI, Wanfang, VIP, and CBM databases. |
| 11 | Search software used | No special search software was used. |
| 12 | Use of hand searching | Hand searching of references cited in final 24 included studies was performed. |
| 13 | List of citations located and excluded | The number of records identified, excluded, and included at each step is diagrammed in the PRISMA flow chart (Figure 1), with reasons for exclusion provided. |
| 14 | Method of addressing non-English articles | Both English and Chinese articles were included (via multilingual database searching). |
| 15 | Method of handling abstracts and unpublished studies | Abstracts and unpublished studies were not included. |
| 16 | Description of any contact with authors | No contact with authors was made. |
| Reporting of methods should include | | |
| 17 | Relevance of assembled studies to the hypothesis | All included studies reported at least one outcome related to the efficacy of auriculotherapy in CHF. |
| 18 | Rationale for data selection and coding | Variables were selected based on pre-designed data extraction forms, including study characteristics, intervention measures, and outcome indicators. |
| 19 | Documentation of data classification and coding | Data was independently extracted by two researchers, with inconsistencies resolved by a third reviewer. Data coding was consistent with the methodology and definitions of the original studies. |
| 20 | Assessment of confounding | When the extracted data was found in median and range, the mean and standard deviation was estimated as described. |
| 21 | Assessment of study quality | Study quality was assessed using the Cochrane Collaboration risk of bias assessment tool, with results reported in Figure 2. |
| 22 | Assessment of heterogeneity | Heterogeneity was assessed using I² statistic and P-value. Visual assessment of Forest plots was also performed. |
| 23 | Description of statistical methods | Meta-analysis was performed using RevMan 5.4. Effect sizes included relative risk (RR), mean difference (MD), and standardized mean difference (SMD) with 95% confidence intervals (CI). Fixed-effect model was used for low heterogeneity (P≥0.1, I²≤50%); random-effects model for high heterogeneity (P<0.1, I²>50%). |
| 24 | Provision of appropriate tables and graphics | A PRISMA flow chart (Figure 1), Table 1 (baseline characteristics of included studies), and Forest plots for outcomes (Figure 3, 4 and Supplementary Figure 1,2,3) were provided. |
| Reporting of results should include | | |
| 25 | Graphic summarizing individual and overall estimates | Forest plots for each outcome (cardiac function improvement rate, LVEF, LVEDD, 6MWT, MLHFQ, adverse reactions) are provided in Figure 3, 4 and Supplementary Figure 1,2,3 |
| 26 | Table with descriptive information for each study | Baseline characteristics of included studies (year, sample size, age, intervention, etc.) are reported in Table 1. |
| 27 | Results of sensitivity testing | Sensitivity analysis was performed by sequentially excluding individual studies for outcomes with high heterogeneity, showing stable results after excluding heterogeneous studies. |
| 28 | Indication of statistical uncertainty | 95% CIs are presented with all estimates of overall effect (OR, MD). |
| Reporting of discussion should include | | |
| 29 | Quantitative assessment of bias | The discussion addressed publication bias (evaluated via funnel plot, Supplementary Figure 4) and risk of bias within studies (assessed by Cochrane tool). |
| 30 | Justification for exclusion | Studies were excluded if they were non-RCTs, duplicates, had incomplete data, or were unrelated to auriculotherapy for CHF, as described in the methods section. |
| 31 | Assessment of quality of included studies | The quality of included studies was discussed, potential limitations in blinding and standardization. |
| Reporting of conclusions should include | | |
| 32 | Consideration of alternative explanations | The discussion addressed potential sources of heterogeneity (e.g., differences in intervention protocols, treatment duration) and their impact on results. |
| 33 | Generalization of the conclusions | The results are generalizable to patients with CHF, supporting auriculotherapy as a supplementary treatment to improve cardiac function and quality of life, with good safety. |
| 34 | Guidelines for future research | Future studies should focus on rigorously designed RCTs with standardized auriculotherapy protocols, sham controls, long-term follow-up, and exploration of optimal stimuli and frequencies. |
| 35 | Disclosure of funding source | This study received no funding. |

Supplementary Table 3: Search Strategy

| Pubmed | | |
| --- | --- | --- |
| Search number | Query | Results |
| 11 | (((("Auriculotherapy"[Mesh]) OR ("Acupuncture"[Mesh] OR "Acupuncture Therapy"[Mesh] OR "Acupuncture, Ear"[Mesh])) OR (((((((((((((((Acupunctures, Ear[Title/Abstract]) OR (Ear Acupunctures[Title/Abstract])) OR (Acupuncture, Auricular[Title/Abstract])) OR (Acupunctures, Auricular[Title/Abstract])) OR (Auricular Acupunctures[Title/Abstract])) OR (Auricular Acupuncture[Title/Abstract])) OR (Ear Acupuncture[Title/Abstract])) OR (Auriculotherapies[Title/Abstract])) OR (Acupuncture Treatment[Title/Abstract])) OR (Acupuncture Treatments[Title/Abstract])) OR (Treatment, Acupuncture[Title/Abstract])) OR (Therapy, Acupuncture[Title/Abstract])) OR (Acupotomy[Title/Abstract])) OR (Acupotomies[Title/Abstract])))) AND (("Randomized Controlled Trial" [Publication Type]) OR ((randomized[Title/Abstract]) OR (placebo[Title/Abstract])))) AND (("Heart Failure"[Mesh]) OR ((((((((((((((Myocardial Failure[Title/Abstract]) OR (Left Sided Heart Failure[Title/Abstract])) OR (Left-Sided Heart Failure[Title/Abstract])) OR (Heart Failure, Left Sided[Title/Abstract])) OR (Heart Failure, Left-Sided[Title/Abstract])) OR (Right Sided Heart Failure[Title/Abstract])) OR (Right-Sided Heart Failure[Title/Abstract])) OR (Heart Failure, Right Sided[Title/Abstract])) OR (Heart Failure, Right-Sided[Title/Abstract])) OR (Heart Failure, Congestive[Title/Abstract])) OR (Congestive Heart Failure[Title/Abstract])) OR (Decompensation, Heart[Title/Abstract])) OR (Heart Decompensation[Title/Abstract])) OR (Cardiac Failure[Title/Abstract]))) | 10 |
| 10 | (("Auriculotherapy"[Mesh]) OR ("Acupuncture"[Mesh] OR "Acupuncture Therapy"[Mesh] OR "Acupuncture, Ear"[Mesh])) OR (((((((((((((((Acupunctures, Ear[Title/Abstract]) OR (Ear Acupunctures[Title/Abstract])) OR (Acupuncture, Auricular[Title/Abstract])) OR (Acupunctures, Auricular[Title/Abstract])) OR (Auricular Acupunctures[Title/Abstract])) OR (Auricular Acupuncture[Title/Abstract])) OR (Ear Acupuncture[Title/Abstract])) OR (Auriculotherapies[Title/Abstract])) OR (Acupuncture Treatment[Title/Abstract])) OR (Acupuncture Treatments[Title/Abstract])) OR (Treatment, Acupuncture[Title/Abstract])) OR (Therapy, Acupuncture[Title/Abstract])) OR (Acupotomy[Title/Abstract])) OR (Acupotomies[Title/Abstract]))) | 35,125 |
| 9 | ((((((((((((((Acupunctures, Ear[Title/Abstract]) OR (Ear Acupunctures[Title/Abstract])) OR (Acupuncture, Auricular[Title/Abstract])) OR (Acupunctures, Auricular[Title/Abstract])) OR (Auricular Acupunctures[Title/Abstract])) OR (Auricular Acupuncture[Title/Abstract])) OR (Ear Acupuncture[Title/Abstract])) OR (Auriculotherapies[Title/Abstract])) OR (Acupuncture Treatment[Title/Abstract])) OR (Acupuncture Treatments[Title/Abstract])) OR (Treatment, Acupuncture[Title/Abstract])) OR (Therapy, Acupuncture[Title/Abstract])) OR (Acupotomy[Title/Abstract])) OR (Acupotomies[Title/Abstract])) | 6,508 |
| 8 | ("Randomized Controlled Trial" [Publication Type]) OR ((randomized[Title/Abstract]) OR (placebo[Title/Abstract])) | 1,157,810 |
| 7 | (randomized[Title/Abstract]) OR (placebo[Title/Abstract]) | 922,278 |
| 6 | "Randomized Controlled Trial" [Publication Type] | 651,133 |
| 5 | "Acupuncture"[Mesh] OR "Acupuncture Therapy"[Mesh] OR "Acupuncture, Ear"[Mesh] | 33,255 |
| 4 | "Auriculotherapy"[Mesh] | 686 |
| 3 | ("Heart Failure"[Mesh]) OR ((((((((((((((Myocardial Failure[Title/Abstract]) OR (Left Sided Heart Failure[Title/Abstract])) OR (Left-Sided Heart Failure[Title/Abstract])) OR (Heart Failure, Left Sided[Title/Abstract])) OR (Heart Failure, Left-Sided[Title/Abstract])) OR (Right Sided Heart Failure[Title/Abstract])) OR (Right-Sided Heart Failure[Title/Abstract])) OR (Heart Failure, Right Sided[Title/Abstract])) OR (Heart Failure, Right-Sided[Title/Abstract])) OR (Heart Failure, Congestive[Title/Abstract])) OR (Congestive Heart Failure[Title/Abstract])) OR (Decompensation, Heart[Title/Abstract])) OR (Heart Decompensation[Title/Abstract])) OR (Cardiac Failure[Title/Abstract])) | 196,662 |
| 2 | (((((((((((((Myocardial Failure[Title/Abstract]) OR (Left Sided Heart Failure[Title/Abstract])) OR (Left-Sided Heart Failure[Title/Abstract])) OR (Heart Failure, Left Sided[Title/Abstract])) OR (Heart Failure, Left-Sided[Title/Abstract])) OR (Right Sided Heart Failure[Title/Abstract])) OR (Right-Sided Heart Failure[Title/Abstract])) OR (Heart Failure, Right Sided[Title/Abstract])) OR (Heart Failure, Right-Sided[Title/Abstract])) OR (Heart Failure, Congestive[Title/Abstract])) OR (Congestive Heart Failure[Title/Abstract])) OR (Decompensation, Heart[Title/Abstract])) OR (Heart Decompensation[Title/Abstract])) OR (Cardiac Failure[Title/Abstract]) | 62,971 |
| 1 | "Heart Failure"[Mesh] | 162,430 |
|  |  |  |
| Embase | | |
| Search number | Query | Results |
| #10 | #3 AND #6 #9 | 195 |
| #9 | #7 OR #8 | 2018573 |
| #8 | randomized OR placebo:ab,ti | 2016473 |
| #7 | 'randomized controlled trial'/exp OR 'randomized controlled trial' OR (randomized AND controlled AND ('trial'/exp OR trial)) | 1542536 |
| #6 | #4 OR #5 | 83657 |
| #5 | 'acupunctures, ear' OR (acupunctures, AND ('ear'/exp OR ear)) OR 'ear acupunctures':ab,ti OR 'acupuncture, auricular':ab,ti OR 'acupunctures, auricular':ab,ti OR 'auricular acupunctures':ab,ti OR 'ear acupuncture':ab,ti OR auriculotherapies:ab,ti OR 'acupuncture treatment':ab,ti OR 'acupuncture treatments':ab,ti OR 'treatment, acupuncture':ab,ti OR 'therapy, acupuncture':ab,ti OR 'pharmacoacupuncture treatment':ab,ti OR 'treatment, pharmacoacupuncture':ab,ti OR 'phamacoacupuncture therapy':ab,ti OR 'therapy, pharmacoacupuncture':ab,ti OR acupotomy:ab,ti OR acupotomies:ab,ti OR pharmacopuncture:ab,ti OR auriculotherapy:ab,ti OR 'acupuncture therapy':ab,ti OR acupuncture:ab,ti OR 'auricular acupuncture therapy':ab,ti OR 'auricular acupuncture':ab,ti | 46746 |
| #4 | 'acupuncture'/exp | 83594 |
| #3 | #1 OR #2 | 1076315 |
| #2 | 'heart failure'/exp | 1055077 |
| #1 | 'cardiac failure'/exp OR 'cardiac failure' OR (('cardiac'/exp OR cardiac) AND ('failure'/exp OR failure)) OR 'heart decompensation':ab,ti OR 'decompensation, heart':ab,ti OR 'congestive heart failure':ab,ti OR 'heart failure, congestive':ab,ti OR 'heart failure, right-sided':ab,ti OR 'heart failure, right sided':ab,ti OR 'right-sided heart failure':ab,ti OR 'right sided heart failure':ab,ti OR 'heart failure, left-sided':ab,ti OR 'heart failure, left sided':ab,ti OR 'left-sided heart failure':ab,ti OR 'left sided heart failure':ab,ti OR 'myocardial failure':ab,ti | 927381 |
|  |  |  |
| Cochrane library | | |
| Search number | Query | Results |
| #1 | MeSH descriptor: [Heart Failure] explode all trees | 15105 |
| #2 | Cardiac Failure or Heart Decompensation or Decompensation, Heart or Congestive Heart Failure or Heart Failure, Congestive or Heart Failure, Right-Sided or Heart Failure, Right Sided or Right-Sided Heart Failure or Right Sided Heart Failure or Heart Failure, Left-Sided or Heart Failure, Left Sided or Left-Sided Heart Failure or Left Sided Heart Failure or Myocardial Failure | 38042 |
| #3 | #1 or #2 | 44510 |
| #4 | MeSH descriptor: [Acupuncture] explode all trees | 216 |
| #5 | MeSH descriptor: [Auriculotherapy] explode all trees | 344 |
| #6 | Acupunctures, Ear or Ear Acupunctures or Acupuncture, Auricular or Acupunctures, Auricular or Auricular Acupunctures or Auricular Acupuncture or Ear Acupuncture or Auriculotherapies or Acupuncture Treatment or Acupuncture Treatments or Treatment, Acupuncture or Therapy, Acupuncture or Pharmacoacupuncture Treatment or Treatment, Pharmacoacupuncture or Phamacoacupuncture Therapy or Therapy, Pharmacoacupuncture or Acupotomy or Acupotomies or Pharmacopuncture | 20144 |
| #7 | #4 or #5 or #6 | 20199 |
| #8 | randomized controlled trial OR randomized OR placebo | 1483428 |
| #9 | #3 and #7 and #8 | 99 |
|  |  |  |
| CNKI | | |
|  | （篇关摘：心衰(模糊)）OR（篇关摘：心力衰竭(模糊)）OR（篇关摘：心脏衰竭(模糊)）OR（篇关摘：心肌衰竭(模糊)）OR（篇关摘：心脏代偿失调(模糊)）AND（篇关摘：耳穴疗法(模糊)）OR（篇关摘：耳穴(模糊)）OR（篇关摘：耳针(模糊)）OR（篇关摘：耳刺(模糊)）OR（篇关摘：针刺(模糊)）OR（篇关摘：针刺疗法(模糊)）OR（篇关摘：穴位按压(模糊)）OR（篇关摘：穴位(模糊)）AND（篇关摘：随机对照试验(模糊)）OR（篇关摘：随机对照(模糊)）OR（篇关摘：随机(模糊)）OR（篇关摘：RCT(模糊)） | 305 |
|  |  |  |
| Wangfang | | |
|  | 主题:(心衰 or 心力衰竭 or 心肌衰竭 or 心脏衰竭 or 心脏代偿失调) and 主题:(耳穴疗法 or 耳穴 or 耳针 or 耳刺 or 针刺 or 针刺疗法 or 穴位 or 穴位按压) and 摘要:(随机对照试验 or 随机对照 or 随机 or RCT) | 479 |
|  |  |  |
| VIP | | |
|  | [((((((题名或关键词=心衰 OR 题名或关键词=心力衰竭) OR 题名或关键词=心肌衰竭) OR 题名或关键词=心脏衰竭) OR 题名或关键词=心脏代偿失调) AND (((((((题名或关键词=耳穴疗法 OR 题名或关键词=耳穴) OR 题名或关键词=耳针) OR 题名或关键词=耳刺) OR 题名或关键词=针刺) OR 题名或关键词=针刺疗法) OR 题名或关键词=穴位) OR 题名或关键词=穴位按压)) AND (((摘要=随机对照试验 OR 摘要=随机对照) OR 摘要=随机) OR 摘要=RCT))](https://qikan.cqvip.com/Qikan/search/index?LngMySearHistoryIdGuid=e110350e-e71b-4565-ac01-3433e42d269b&from=Qikan_Article_History) | 270 |
|  |  |  |
| CBM | | |
| 序号 | 检索表达式 | 结果 |
| 10 | (#3) OR (#6) OR (#9) | 382 |
| 9 | (#7) OR (#8) | 2420161 |
| 8 | "随机"[常用字段:智能] OR "随机对照"[常用字段:智能] OR "RCT"[常用字段:智能] | 2419924 |
| 7 | "随机对照试验"[不加权:扩展] | 204200 |
| 6 | (#4) OR (#5) | 283557 |
| 5 | "针刺疗法"[常用字段:智能] OR "针刺"[常用字段:智能] OR "耳针"[常用字段:智能] OR "耳刺"[常用字段:智能] OR "耳穴"[常用字段:智能] OR "穴位"[常用字段:智能] OR "穴位按压"[常用字段:智能] | 283556 |
| 4 | "耳穴疗法"[不加权:扩展] | 1134 |
| 3 | (#1) OR (#2) | 428206 |
| 2 | "心衰"[常用字段:智能] OR "心脏衰竭"[常用字段:智能] OR "心肌衰竭"[常用字段:智能] OR "心脏代偿失调"[常用字段:智能] | [42](javascript:historyLink(')8206 |
| 1 | "心力衰竭"[不加权:扩展] | [23](javascript:historyLink(')9778 |

Supplementary Figure 1:Meta-analysis of auricular acupoint therapy on LVEDD in patients with chronic heart failure


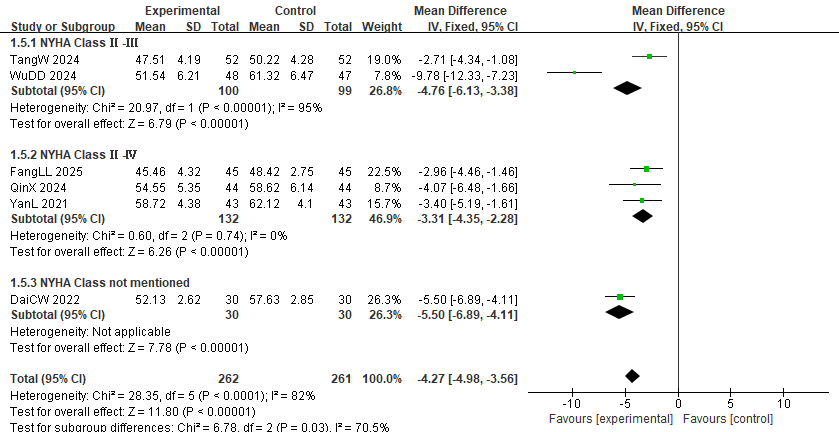


Supplementary Figure 2:Meta-analysis of auricular acupoint therapy on 6MWT in patients with chronic heart failure


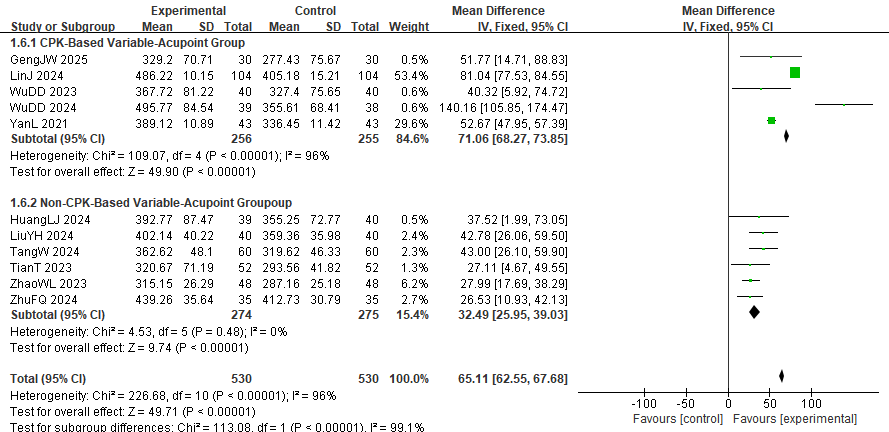


Supplementary Figure 3:Meta-analysis of auricular acupoint therapy on MLHFQ in patients with chronic heart failure


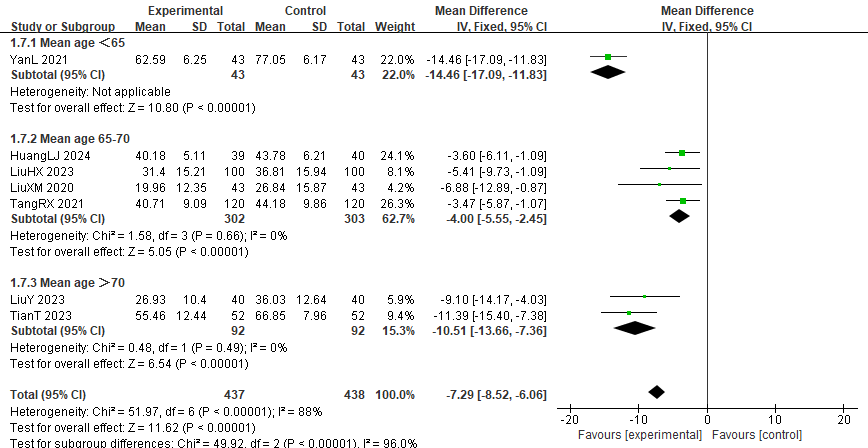


Supplementary Figure 4:A: funnel plot of total effective rate; B: funnel plot of LVEF; C: funnel plot of 6MWT


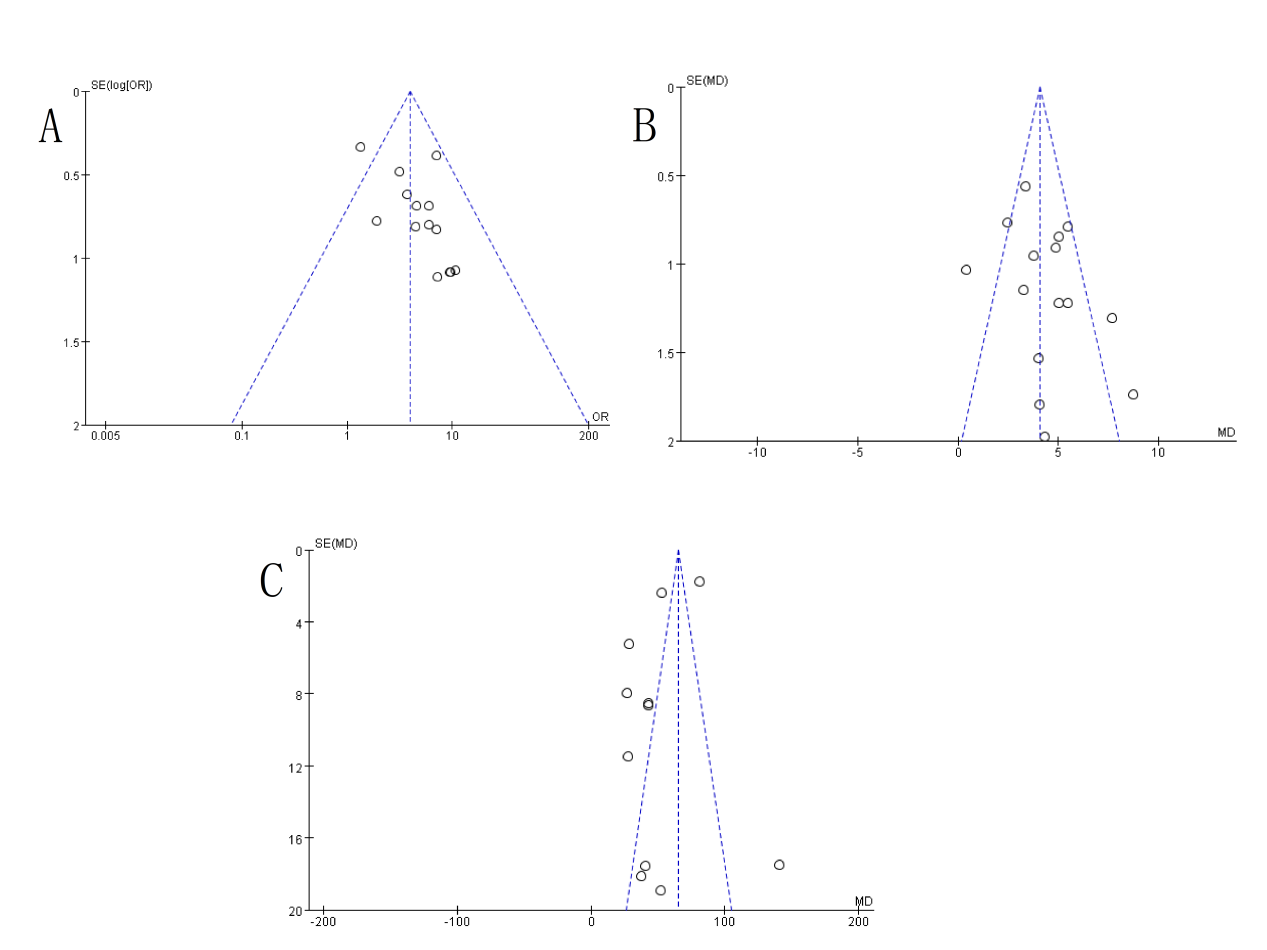

Supplement: Supplementary file 1 [file Datasheet1.docx]
